# The IGS Standard Operating Procedure for Automated Prokaryotic Annotation

**DOI:** 10.4056/sigs.1223234

**Published:** 2011-04-25

**Authors:** Kevin Galens, Joshua Orvis, Sean Daugherty, Heather H. Creasy, Sam Angiuoli, Owen White, Jennifer Wortman, Anup Mahurkar, Michelle Gwinn Giglio

**Affiliations:** Institute for Genome Sciences, University of Maryland School of Medicine, Baltimore, MD, USA

**Keywords:** Institute for Genome Sciences, functional annotation, structural annotation, microbial genomics, prokaryotic genomics, annotation pipeline, pFunc, Glimmer, HMM, BER, Ergatis, Manatee, IGS Annotation Engine

## Abstract

The Institute for Genome Sciences (IGS) has developed a prokaryotic annotation pipeline that is used for coding gene/RNA prediction and functional annotation of *Bacteria* and *Archaea*. The fully automated pipeline accepts one or many genomic sequences as input and produces output in a variety of standard formats. Functional annotation is primarily based on similarity searches and motif finding combined with a hierarchical rule based annotation system. The output annotations can also be loaded into a relational database and accessed through visualization tools.

## Introduction

**Figure 1:**
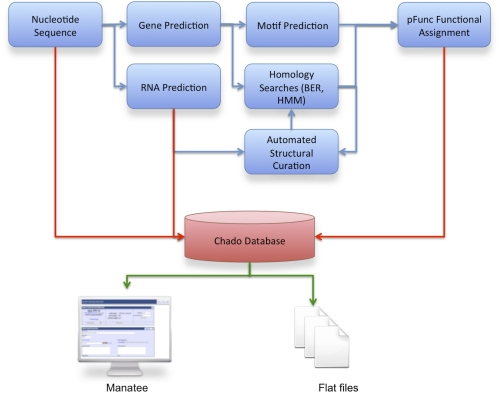
Flow of data and logic for IGS automated microbial annotation pipeline. Protein coding genes and RNAs are predicted from nucleotide sequence, which are then structurally curated and assigned a function.

The IGS prokaryotic annotation pipeline can be used for the annotation of *Bacteria* and *Archaea*. This pipeline forms the core of the IGS Annotation Engine [1], a free annotation service for prokaryotic sequences. It is also used as the annotation system for prokaryotes sequenced under the IGS Genome Sequencing Center for Infectious Disease [2]. The IGS prokaryotic annotation pipeline can be applied to both draft and finished sequences and has been successfully used in the annotation of hundreds of genomes. The pipeline includes gene finding, protein searches, and the pFunc evidence hierarchy that produces automated functional annotation. The output of this pipeline can be stored in a Chado [3] relational database and can be accessed with Manatee [4] for annotation visualization and curation (Figure 1). Output of the pipeline is also available in a variety of flat file formats. The pipeline is managed using the Ergatis [5] framework and is available on Sourceforge.

## Requirements

The IGS prokaryotic annotation pipeline accepts a multi-sequence nucleotide fasta file as input. Annotation can also be performed on an existing set of gene predictions, which simply skips the structural prediction steps of coding sequences. In addition, the name and locus tag prefix (if applicable) of the organism are also required. Structural prediction is performed on the input sequences, followed by similarity searches against public datasets. The final steps of the pipeline include running polypeptide analysis tools as well as automated functional annotation. The output is then converted to various output formats as required. The pipeline uses open source or free software whenever possible. All unique tools written specifically for the pipeline are written in PERL and distributed under the GNU public license on the Ergatis Sourceforge website.

## Procedure

### Structural Annotation

The pipeline starts by splitting the multi-sequence nucleotide fasta file into individual files. Non-coding RNA and protein coding genes are predicted first, in parallel on each input sequence.

### Non-coding RNA Structural Annotation

Non-coding RNA genes are predicted using RNAmmer [6] and tRNA-scanSE [7]. RNAmmer predicts rRNA genes (5s, 16s, and 23s) using the standard HMM dataset distributed with the software. Transfer RNA genes are predicted using tRNA-scanSE using default values except where specifying the organism type (bacteria are selected by default for the pipeline).

### Coding Gene Structural Annotation

Protein coding genes are predicted using a self-training method with Glimmer3 [8]. A set of non-overlapping long ORFs is produced from the input nucleotide sequences and used as a training set to further refine the gene structural predictions in a second iteration of Glimmer3. An upstream position weight matrix is created and aids in identifying ribosomal binding sites. In addition, the relative frequency of start sites is calculated. The position weight matrix and start site frequencies, along with the original long ORFs training set, are used as input into the second Glimmer3 iteration. The results from the second run are used as the working prediction and this set is automatically curated later in the pipeline.

### Similarity Searches (Round 1)

An initial blastx [9] is run against UniRef100 [10] to generate first pass pairwise alignments. These pairwise alignments are then used as input into BER [11] (**B**last **E**xtend **R**epraze). BER is a modified Smith-Waterman [12] algorithm that aligns an extended query nucleotide sequence against a protein match. The nucleotide query sequence (including extensions of 300 nucleotides upstream and 300 nucleotides downstream) are translated and aligned to each protein match from the blastx analysis resulting in up to 150 alignments. Including the extensions in the alignment aids in the detection of potential sequencing errors or mutations that may result in frameshifts or in-frame stop codons. Once a region of alignment is detected, the BER tool is able to extend the alignment through potential frame shifts or in-frame stop codons. These extensions allow such alignments to continue past the original boundaries of the predicted gene, thus enabling better curation of the gene models. In essence, BER shows similarity between sequences beyond gene boundaries. Only one round of extensions is performed. Further manual assessment of flanking regions must be employed to resolve regions of similarity that extend beyond 300 nucleotides upstream or downstream of the predicted gene. BER matches are evaluated and ranked as described in the Functional Annotation section below and in Table 1. 

**Table 1:** BER annotation hierarchy

**Trusted**	**Query % Cov.**	**Match % Cov.**	**Rank**	**Name Modifier**	**GO Terms/ TIGR roles**
Yes	Full	Full	1	None	copied from match
Yes	Full	Partial	2	… domain protein	GO root terms/TIGR unknown
Yes	Partial	Full	2	… domain protein	copied from match
No	Full	Full	3	possible …	copied from match
No	Partial	Full	4	possible …domain protein	GO root/TIGR unknown
No	Full	Partial	4	possible…domain protein	GO root/TIGR unknown
with ambiguous term	Full/Partial	Full/Partial	5	“conserved hypothetical protein”	GO root/TIGR conserved hypothetical

The HMMER package [13] is then used to search the predicted polypeptides against two databases: TIGRFams [14] and PFams [15]. The output of the HMM search is used in the automated structural curation as well as functional annotation portions of the pipeline.

### Automated Similarity-Based Structural Curation

In order to refine the gene predictions, a round of automated evidence-directed structural curation is performed. The first step is to evaluate the start sites of predicted genes. There can be multiple potential start sites in the upstream region of an open reading frame and BER sequence alignments can be used to give us a better idea of the correct start site. For this, we run a start site curation tool, which uses a voting based algorithm to determine the most likely start site for a particular open reading frame. The top BER alignments are considered and if the start of a match protein aligns with a start site in the query, this is counted as a vote. The upstream regions of potential start sites are also compared against a simple consensus sequence to determine if a ribosomal binding site is likely to be present. If the consensus sequence matches the region upstream, this is also considered as a vote. At the end of the algorithm, the start site with the most votes is kept. In the case of a tie, the BER matches with the best p-values are weighted more than other evidence. In the majority of cases, this agrees with the Glimmer3-called start site.

In order to identify false positive gene predictions, all overlapping genes (both ncRNA and protein coding) are identified. This is especially necessary in genomes with higher GC content, due to the lower frequency of stop codons, resulting in an increased frequency of long, random open reading frames. When an overlap of greater than 60 base pairs is found, both genes involved in the overlap are evaluated with respect to evidence from BER and HMM. If a gene with no evidence overlaps a gene with evidence, this suggests that the former is a false positive. That gene is then removed from the predicted set. If an overlap of greater than 60 base pairs is found between a predicted RNA and a gene with no evidence, the gene is removed from the prediction set. All other overlaps of greater than 60 base pairs are flagged for manual review.

There are possible false negatives in the gene prediction at this point. In order to reduce the frequency of these errors, we search interevidence regions against UniRef100 using blastx. Interevidence regions can be defined as contiguous sequences of intergenic regions and predicted genes that do not contain any evidence from BER or HMM searches. The pairwise alignments produced by blastx are available for manual review and genes can be added where appropriate.

### Similarity Searches (Round 2)

After the automatic curation of start sites, the newly changed gene models are retranslated. These new polypeptides are then run through another set of blastx, HMM and BER searches to update similarity evidence for functional annotation.

### Motif Prediction

Each polypeptide is run through a set of motif prediction tools. SignalP [16] is used to predict the existence and location of signal peptide cleavage sites and LipoP [17] is used to predict the existence of lipoprotein signal peptides. TMHMM [18] is used to predict transmembrane helices. Each polypeptide is also scanned with PROSITE [19] using ScanProsite to identify consensus patterns that are indicative of binding sites, active sites, etc. The -s option is used in order to skip the frequently matching, unspecific patterns. In addition, each polypeptide is run against the NCBI COGs [20] dataset. Finally, each polypeptide is searched against the Priam [21] dataset using reverse PSI-Blast (RPS-Blast) [22] in order to aid in the assignment of EC numbers [23].

## Functional Annotation

The functional annotation portion of the pipeline uses a combination of sequence similarity searches and other bioinformatics tools to assign a common name, a gene symbol, GO terms [24], EC numbers and TIGR roles to each polypeptide. These annotations are assigned by the program pFunc (prokaryotic **p**rotein **func**tional prediction.) pFunc is a modular tool which parses various evidence types and filters this set based on a set of cutoffs. The program then applies an evidence hierarchy to all available information to assign the best possible annotation for each polypeptide. The current implementation of the pipeline uses information from BER, HMM, LipoP and TMHMM searches to assign a common name, a gene symbol, EC numbers, GO terms and TIGR roles to each polypeptide, as applicable. pFunc first evaluates each evidence type individually to choose the best annotation for that type.

### BER

Matches that show less than 40% identity are removed from consideration for annotation. Each remaining match is then evaluated to determine if it is considered trusted. Trusted matches are those which a) have been characterized through experimental means (usually determined from the literature) b) are considered by Uniprot to have experimental evidence confirming annotated function or c) were annotated in a GO association file using an experimental evidence code (EXP, IDA, IPI, IMP, IGI, IEP.) These types of matches are considered more reliable than other, non-trusted BER matches.

The percent coverage for both the query and match proteins is also considered when determining the best BER match for functional annotation. A cutoff score of 80% coverage is used to determine partial vs full matches. Coverage is considered separately for both query and match proteins. For example, a BER match with 85% coverage of the query protein and 75% of the match protein would be considered a “full query, partial match” alignment.

Any non-trusted BER matches that contain ambiguous terms (e.g. putative, probable) in the common name are replaced with “conserved hypothetical protein” and the root GO terms, as well as the TIGR role, are assigned as conserved hypothetical proteins. The best BER match is chosen from the remaining set following the hierarchy in Table 1.

### HMM

Each HMM is considered separately, based on the isology types of HMM and also the individual cutoff scores. Any HMM match that does not pass trusted cutoff is not considered for annotation. The best annotation from the HMM set of evidence is chosen at this stage and a suffix is appended to the end of the common name depending on the isology as seen in Table 2. With the exception of the “Pfam” isology type, all isologies included in this hierarchy are from TIGRfams.

**Table 2:** HMM annotation hierarchy.*

**Isology**	**Rank**	**Name Modifier**
Equivalog	1	None
Equivalog Domain	2	None
Subfamily	3	… family protein
Superfamily	4	… family protein
Subfamily Domain	5	… domain protein
Domain	6	… domain protein
Pfam	7	… family protein
Hypothetical Equivalog	7	None

*In all cases, GO terms and TIGR roles are copied from the HMM.

### LipoP and TMHMM

LipoP (lipoprotein predictions) are also considered when assigning annotations. Polypeptides containing a LipoP prediction but no BER or HMM evidence will be annotated with the common name “putative lipoprotein”, GO term component: membrane (GO:0016020) and the TIGR role “cell envelope: other” (88).

A polypeptide is considered for annotation by TMHMM when it has 5 or more predicted membrane-spanning regions. When this occurs, the annotation from TMHMM is considered. The annotation is the same as that from LipoP with the exception of the common name, “putative integral membrane protein”

### pFunc

Following the parsing and initial filtering of possible annotations, pFunc will apply a final annotation hierarchy to the set of best annotations provided by the previous steps. See Table 3 for the hierarchy. Any protein not containing evidence from one of the 18 ranks will be called “hypothetical protein” and assigned the GO root terms and the TIGR role id for “hypothetical protein.” In the rest of the cases, the annotation will be transferred directly from the top-scoring evidence based on the hierarchy in Table 3. 

**Table 3:** Final annotation hierarchy

**Evidence**	**Criterion**	**Query**	**Match**	**Rank**
HMM	Equivalog	N/A	N/A	1
BER	Trusted	Full	Full	2
HMM	Equivalog Domain	Full	Full	3
BER	Trusted	Partial	Full	4
HMM	Subfamily	N/A	N/A	5
HMM	Superfamily	N/A	N/A	6
HMM	Subfamily Domain	N/A	N/A	7
HMM	Domain	Partial	Full	8
HMM	Pfam	Full	Full	9
BER	Trusted	Full	Partial	10
TMHMM	> 5 membrane spans	N/A	N/A	11
LipoP	Presence of prediction	N/A	N/A	12
HMM	Hypothetical Equivalog	N/A	N/A	13
BER	Not trusted	Full	Full	14
BER	Not trusted	Partial	Full	15
BER	Not trusted	Full	Partial	16
BER	With ambiguous term	Full/Partial	Full/Partial	17

### Functional Annotation Post-Processing

Post-processing is necessary to verify common names, assign additional information and fix common mistakes when automatically assigning annotation. Nonsensical common names can often result when appending various suffixes depending on annotation type. These types of errors are corrected by changing suffixes to fit accordingly. In addition, the common names are searched for other assertions (i.e. gene symbols, EC numbers) present from transferring names from public datasets, which are then moved to the proper location. EC numbers are not modified during this step and partial EC numbers are left as valid annotations. The common names are also scanned for functional keywords and assigned high-level TIGR roles based on these keywords if no other role has been assigned.

## Output Formats

The IGS prokaryotic annotation pipeline supports various output formats. Initially, an XML representation of the nucleotide sequences and annotation is generated. Each gene (ncRNA and protein coding) is assigned a locus tag using the input locus tag prefix. The genes are numbered sequentially, starting with the first predicted gene of the longest input nucleotide sequence.

The XML can be automatically reformatted into tbl, asn or Genbank formats. The XML representation is often used to load a Chado database for use with the manual annotation tool Manatee. Through this interface, tab files, CDS sequence files, polypeptide sequence files, Genbank and GO annotation files can be generated.

**Future Development**

Further development is planned for capturing more complex protein functions in annotations. Currently, since annotation is only transferred from the top-scoring source, bifunctional or multifunctional genes will only receive one function assignment automatically. In many cases, this will also be annotated as a “domain protein”.  Future work will involve developing a strategy to detect bifunctional proteins and assign them annotations as such.

Another area for future development is handling multiple copies of a gene within a genome. Currently, the pipeline will not detect the assignment of the same gene symbol to multiple genes. In the future, a system that evaluates the relative strengths of the evidence for each gene with the same gene symbol could be put into place. The gene with the most functional evidence will be assigned the gene symbol and all other instances of that gene symbol in the genome will be removed.

Finally, we plan to automatically flag genes that have putative frameshifts or in-frame stop codons based on the presence of such features in BER alignments and produce a report for manual review.

## Implementation

The IGS prokaryotic annotation pipeline is implemented as a template inside of the Ergatis workflow system. Each step is run in parallel where it makes sense to do so. If not otherwise specified, each of the steps is written in the PERL programming language. Table 4 shows the versions and parameters of third-party software used in the pipeline.

**Table 4:** Software versions and parameters

**Section**	**Tool**	**Version**	**Parameters**
Structural	tRNA-scanSE	1.23	-q -b -B
Annotation	RNAmmer	1.2	-S bac -m lsu,tsu,ssu, -xml -gff
	Glimmer3	3.02	-o50 -g110 -t30 -z11 -l -X
Functional	blastall -p blastx	2.2.17	-e 1e-5 -F T -b 150 -v 150 -M BLOSUM62
Annotation	HMMer	2.3.2	-acc
	SignalP	3.0b	-m ‘nn+hmm’ -trnc 1000 -graphics ‘gif+eps’
	TMHMM	2.0c	--libdir TMHMM/lib
	LipoP	1.0a	-short -cutoff -3
	Prosite (ps_scan)	1.34	-s
	RPS-blast	2.2.17	-e 1e-5 -F T -b 150 -v 150
	Blastp	2.2.17	-e 1e-5 -F T -b 150 -v 150 -M BLOSUM62

## Summary

The IGS prokaryotic annotation pipeline has been used for the annotation of hundreds of genomes. It provides gene predictions and automated functional annotation accessible via a Chado relational database and the associated curation tool Manatee or through flat files. The core of the pipeline is the evidence hierarchy of the pFunc software. The general scientific public can have access to the pipeline through the IGS Annotation Engine, which provides free automated annotation for prokaryotic sequences. As additional prediction tools and search databases are developed they will be assessed and added to the pipeline and the pFunc hierarchy as appropriate.
